# Lived experiences of tuberculosis patients and their implications for early tuberculosis case identification and management in pastoralist community setting: a qualitative study in Borena zone, Oromia region of Ethiopia

**DOI:** 10.1186/s12913-020-05787-1

**Published:** 2020-10-09

**Authors:** Abebe Megerso, Negussie Deyessa, Godana Jarso, Alemayehu Worku

**Affiliations:** 1 Department of Public Health, Adama Hospital Medical College, Adama, Ethiopia; 2 Department of Preventive Medicine, School of Public Health, Addis Ababa University, Addis Ababa, Ethiopia; 3 Department of Internal Medicine, Adama Hospital Medical College, Adama, Ethiopia

**Keywords:** Case detection, DOTs, Lived experience, Pastoral, Tuberculosis (TB)

## Abstract

**Background:**

Ethiopia has highly diversified population with notable socioeconomic and cultural differences. Regardless of the differences, short course directly observed treatment,where patients should take drugs under direct observasion of health care providers, is uniformly applied all over the country. Evidences are scarce on how well does this uniform approach fits with the pastoral community setting. The purpose of this study was to explore lived experiences of TB patients in the pastoral community under the uniform approach, and their implications to early case identification and management.

**Method:**

Qualitative method with phenomenological study design was undertaken to explore lived experiences of TB patients. Patients from all levels of health care (hospital, health center and health post) were included. Experience of both drug susceptible and drug resistant TB patients were documented. Twenty one patients, who consented to in the study, were selected by a convenience sampling method. In-depth interview was conducted using a semi-structured interview guide and the interview ended subsequent to information saturation. The interview was audio recorded; and field notes were also taken. Data analysis was done concurrently with the data collection using a word processor designed for qualitative text analysis. InductiveThematic analysis was undertaken to identify key themes.

**Results:**

Twenty one patients (eight from hospitals, nine from health centers and four from health posts) were interviewed. Three of the eight hospital patients were on drug resistant tuberculosis (TB) treatment. Sixty two codes, five code categories and three themes emerged from the interviews. The three themes were health system, stigma and discrimination, and socioeconomic problem related experiences. Inaccessibility to health facilities due to scattered settlement and mobility, delay in care seeking TB symptoms, low index of suspecting TB by care providers, fear of stigma and indirect treatment related costs were some of the codes identified.

**Conclusion:**

TB patients in the pastoral setting were experiencing multifaceted challenges with the current application of ‘*one-size-fits-all’* approach which implied hampered timely case identification and compromised patient management. Therefore, designing context appropriate intervention approach is required to ensure unprejudiced services.

## Background

Pastoralist communities occupy 43% of the land mass of Africa. Ethiopia is one of 36 countries with large area of the pastoralist livelihood community [1]. Pastoral communities live in lowland and dry areas and are mobile, with scattered settlements [2]. Basic infrastructures, including health facilities are scarce and access to the existing few is also difficult is the pastoral settings.

Ethiopia is one of the high tuberculosis (TB), drug resistant TB (DRTB) and TB/HIV burden countries in the world [3, 4]. Directly observed treatment short course (DOTs) is the TB prevention and control strategy in the country over the last two decades [5]. This strategy requires patients to take anti TB medicines under direct observation of health care providers every morning. The country has a highly diversified population, including urbanized community, agrarian and pastoral community with notable socioeconomic and cultural differences [6, 7]. Tuberculosis is a disease determined by various social factors [8, 9]. Cognizant of determinants of TB and need for context appropriate interventions, the World Health Organization (WHO) recommended the socio-cultural context appropriate interventions as one of the required strategies to attain the 2030 ‘End TB’ goals [10]. Ethiopia has adapted the ‘End TB’ strategy and developed a national TB prevention and control guidelines since 2018 [4].

However, the TB prevention and control approach continued to follow the usual DOTs [5] in all parts of the country regardless of differences in socioeconomic and cultural contexts. This ‘one size fits all’ approach is not known to be appropriate for pastoral community [11] and it may marginalize a significant proportion of the country’s population such as the pastoral community. Therefore, with this TB prevention and control approach, it is important to understand lived experiences of TB patients and draw implication of the experiences to the early TB case identification and management in the pastoral communities.

## Methods

### Setting

This study was conducted in a pastoral community of Ethiopia. Pastoralist community covers 60% of land mass and 12–15% of the total population of the country [2, 12, 13]. In Oromia Region pastoralist community constitutes 43% of the land mass and 16% of the regional population [14]. Borena zone is a pastoral community and shares boundary with Kenya in south and Somali regional state in the east.

In this zone, the community is highly mobile for the search of pasture and water for livestock. The community lives in scattered settlements and access to health facilities is limited. Due to their settlement pattern the lowest health facility, health post, is far from most community members. Besides seasonal mobility of the community, house holds can be as far as tens of kilometers from the nearest health facilities and also there is no access to transportation facilities.

### Study design

We applied qualitative method with phenomenological study design to explore lived experiences of TB patients. The phenomenological study helps to describe experiences as they are lived and examines the uniqueness of individual’s lived situations.

### Recruitment strategy

We included patients from all the three treatment levels (hospital, health center and health post) and two treatment categories (TB drug susceptible and drug resistant patients). All patients were on anti TB treatment at least for 2 months before the interview date. Heterogeneous sampling method was used to accommodate facility type variations and convenient sampling for participant selection. At each level, patients were selected for the interview using convenience sampling technique [15, 16]. Every other patient was interviewed in health facilities where TB patient flow was higher, to minimize information contamination which may happen through communication between subsequent patients. The sample size was determined using the emergence of redundant ideas and theoretical information saturation.

### Data collection

Data were collected through in-depth interview using semi-structured interview guides and audio recording during December 01–30, 2019. The interview guide was developed for this study based on initial consultation with TB program experts and clinicians treating TB patients in the study area. Each in-depth interview lasted for 15 to 30 min to explore patients’ lived experiences spanning from date of TB symptoms to the date of interview. Notes were taken to document some non verbal messages at the interview. Interview was conducted by a single interviewer to avoid possible inter interviewer differences. The interviewer has no any relationship with the pastola community. It was conducted in local languages spoken by the participants. After interviewing the first four patients, the interview guide was slightly modified to accommodate new issues emerged from the interviews. The interviewer introduced himself clarifying that he has no any direct responsibility in the health system to minimize social desirability bias of the study participants [17] (Table 1).

**Table 1:** Interview Guides (*Detailed probing questions not included)*

1	Please, tell me what you knew about tuberculosis before you were informed to have been infected with the disease. Mention signs and symptoms, modes of transmission and method of prevention of the disease that you knew before the diagnosis,
2	Tell me how long you stayed at home before visiting a health facility for medical care after having the signs and symptoms of the disease and why.
3	Where did you seek for the first medical care and why you preferred the facility or the place you mentioned?
4	Describe processes you have passed through to reach the first health facility from the day you experienced its signs and symptoms.
5	Describe your experiences regarding the process went through and the challenges of getting treatment for the disease after reaching the health facility. Please, tell me your experiences in any health facility (private or public),
6	Tell me how at ease you are to inform other people in your community that you have TB infection. Please, also tell me the reasons for why or why not at ease.
7	How do you describe your relationship with other people after you were diagnosed to have contracted TB infection. Is there any change in the relationship? If so, why do you think?
8	Based on your own experience, describe conditions that should be changed to make TB treatment approach more suitable for your community setting.

### Data analysis

Data analysis was done concurrently [18] with the data collection using a word processor designed for qualitative text analysis [19]. Interviews conducted on a day were transcribed from the audios over the subsequent night not to miss important notes. Information from the interviews was used to enrich the subsequent interviews. Independent coding was done by two authors to ensure validity and reliability of the themes development [18]. Consistency between the audio and the transcriptions was checked by two of the authors of this article who speak the local language. Audio records and transcripts were cross-checked by independent experts before the final analysis.

Interactions between the interviewer and the interviewee at the data collection, and interaction between the researchers and texts were sources of information in this study. We followed an inductive content analysis approach [20] and identified codes, categories and themes related to our research question. Phenomenological description was used to analyze the manifest content and hermeneutic interpretation to analyze latent contents [17, 20]. Findings were reported using textual descriptions and quotes to illustrate ideas and illuminate experiences [21].

## Results

Twenty one patients who were on anti TB treatment at least for 2 months prior to date of data collection were interviewed across the three levels of health care (eight from hospitals, nine from health centers and four from health posts) (Table 2). Three of the eight hospital patients were on DRTB treatment. Age the patients’ ranges from 22 to 62 years with a median and inter quartile range of 31(26–42) years. From the analysis of interview transcriptions, 62 codes and five categories emanated which were finally organized into three themes. The three emerged include:

- Experiences related to the health systems: Access, care providers’ index of suspicion to presumptive TB,
- Experiences related to stigma and Discrimination towards TB patients and
- Experiences related to the socioeconomic problems: Low TB awareness related prevention and control practices and direct non- medical costs related to TB treatment,

The five categories include: lived experiences related to patients’ TB prevention and control practices, private health facilities related, public health facilities related, stigma and discrimination towards TB patients, and economic problems related experiences.

**Table 2:**
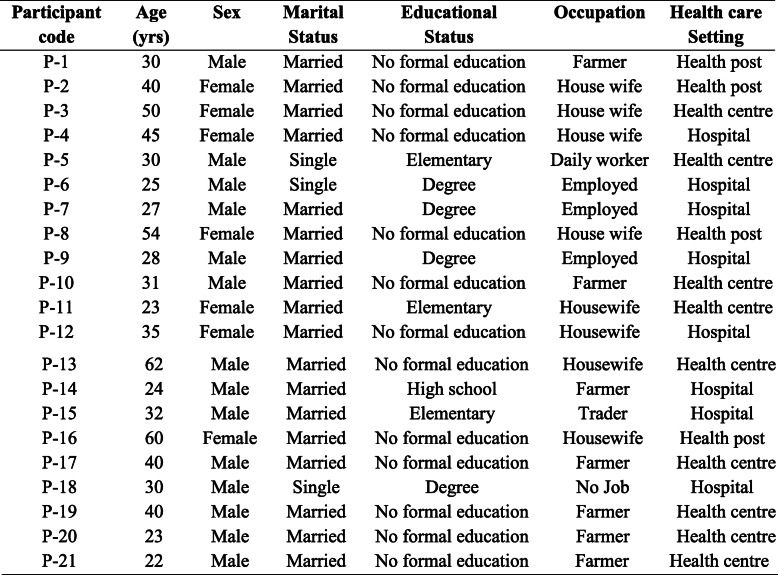
Socio-demographic characteristics of study participants

### Experiences related to the health systems (Table 3)

#### Access to health facilities

Ethiopia’s TB treatment guidelines, the DOTs strategy, required daily visit to health facilities every morning. But, many TB patients cannot go to health facility every morning due to inaccessibility of treatment providing health facilities in the pastoral community. As a solution for this problem, health care providers sometimes decide to give doses of anti TB drugs to patients. The number of doses can be for days or even for weeks and the drugs will be taken at home without having any trained treatment supporter at community level. Inaccessibility of the health facilities and decisions to allow patients to take anti TB drugs at home without having trained treatment supporters affect the disease prevention and control program. Patients can stay in the community for long duration before getting diagnosed and start the treatment and this condition will allow continued transmission of the infection. Inappropriate treatment approach that health care providers choose due to the inaccessibility may affect compliance to the treatment and lead to development of DR TB.

**Table 3:** Key Theme: Experiences related to the health systems

***Sub Themes***	***Quotes from Participants***
Inaccessible Health facility	I brought my four years old child for TB treatment. Our village is far from this hospital and no health facility closer to my home than this. I pay 100 birr per day for motorcycle every morning to come here. I do not have the money to follow this for the whole six months. I want if the doctor tells me how to give the drugs to my child at home and give me his drugs … (**P-15**)
Public Health facilities	*I informed the clinician all the history of my health problem, including treatments I sought. The clinician opted to decide the same way as that of the private clinic. I requested the clinician to check me for TB as I suspected myself of contracting it. He said, ‘I am the one to decide on what to do for patients; no patient urges me to do what the patient needs’. … He gave me some drugs and sent me out. … The disease went worse. I went back to the same hospital and directly went to the office of the CEO and told him all what has happened to me. The CEO advised me to go to same clinician after discussing with the clinician. … The diagnosis turned out to be MDR TB after about six months from the onset of sign and symptom … (****P-9****)*
Private Health facilities	*I went to a private clinic to seek care for loss of appetite, loss of weight and unusual night sweating,* etc. *They said, Your disease is ‘Qora’ meaning ‘cold’ and sent me home with a dozen of oral medications to be taken over a couple of weeks. I took the medications as per their advices but no improvement in my health condition even when I finish the drugs. I went back to the same clinic to inform them of the situation. This time, they changed the diagnosis to ‘Typhoid’ and gave me other types of oral drugs. After a week, I realized that my health is worsening and I went to a nearby hospital …*. (***P-7****)*

NB. (P-7), (P-9) and (P-15) are study participants’ codes given in Table 1

#### Care providers’ index of suspicion to presumptive TB

Many fascinating patients’ lived experiences, related to public health institutions, were revealed in this study. These experiences include a low index of suspecting TB. Health care providers should have higher level of suspicion for TB at least when patients complain signs and symptom of TB not to miss presumptive TB cases. But, this was not the case in both public and private health facilities of the current study setting. Such missed opportunities of TB case identification may render false reassurance to patients as having no TB and cause delay in diagnosis of the disease which in turn leads to continued infection transmission.

#### Private health facility concerns for TB

The other health systems related patients’ experiences were those related to private health facilities practices. Private health facilities discussed in this report were private clinics, pharmacies and drug shops or stores. These facilities are more accessible to the community than the public health institutions in pastoral community setting. Patients buy drugs of their preference and the amount they afford to buy from drug stores. Even in areas where public health facilities are accessible, some patients believe that private clinics provide better health care than public health facilities and prefer to visit them. If these health facilities work to identify TB cases, they can serve as important facilities to identify TB cases earlier. Nonetheless, experience of the current study participants showed that most of such facilities were not good enough to identify TB cases. They squander the time by giving different drugs to patients regardless of identifiable and patient reported symptom complex of TB. Such practices lead to false reassurance to patient as not having TB, delayed case identification and continued disease transmission.

### Experiences related to stigma and discrimination towards TB patients (Table 4)

Stigma and discrimination towards TB patients was one of the themes emerged. It varies from keeping material used by TB patients separated from that of other people until the end of treatment to letting patients live alone in a separate home. Such stigmatization and discrimination of the patients might have emanated from low awareness of the community members. Many of the study participants reported such experiences. They also indicated that there are people who deny having symptom complexes of TB; because their fear the stigma and discrimination. This denial may lead to poor health seeking and affects early TB case identification.

**Table 4:** Key Theme: Experiences related to Stigma and Discrimination towards TB Patient

***Sub Themes***	***Quotes from Participants***
Stigma and discrimination at community level	Since I was known to have TB and taking drugs, people whom I used to live with were not happy to be with me in the same area or same room. I was left to live alone and I did not have a good feeling at that time … (**P-4**)
Stigma and discrimination at work place	But what I observed in my office was like total discrimination of the sick. They tend to leave the whole office when I come in and that has given me a very bad feeling. I believe such practice may affect treatment seeking of other people who may have the disease … (**P-6**)

NB. (P-4) and (P-6) are study participants’ codes given in Table 1

### Experiences related to the socioeconomic problems (Table 5)

Socioeconomic problems, such as lack of access to health information and dependence only on livestock for sources of income, were features that led to experiences that in turn affect early TB patient identification. These problems were exacerbated by the inaccessibility of service providing health facilities in the setting.

**Table 5:** Key Theme: Experiences related to Socioeconomic problems(Low awareness and indirect non-medical cost related to TB Treatment)

***Sub Themes***	***Quotes from Participants***
Awareness severity of the disease	In our community, we consider cough as a simple self limiting problem. If we continue to cough for longer time, we use our traditional remedies such as honey with tea and others that we can prepare at home. Sometimes we buy drugs and use them to get relief … (***P-2****)*
Awareness severity of the disease	TB patients should keep any material she/he used away from other people as the disease can transmit to others until the person gets cured from the disease … … to protect transmission of the disease, I live alone in a separate room from the family. I prefer to live in a rent house in town … (**P-3 and P-19**)
Indirect cost	To get these drugs I start walking as 6:00 AM and reached here, at hospital after over two hours walk. I am sick and difficult for me to walk this long every day. With the capacity I have, I have to till/plough my land in the morning, Now I can not do that as I have to walk to hospital every day and my family is going to face more problem because of my disease. .. … (**P-20**)

NB. (**P-2**) and (**P-3 P-19 and P-20**) are study participants’ codes given in Table 1

#### Low TB awareness related prevention and control practices

Pastoral community does not consider common signs and symptoms of TB as noteworthy conditions. For instance, they call cough as ‘*dofofa*’ which means a mild self-limiting condition. They attribute it to exposure to some unpleasant odor. The community assumes it as a simple self limiting condition and do not seek medical care for such signs and symptoms until every other traditional methods are tried and the problem gets worse. Many participants mentioned points that indicate lack of awareness ranging from considering everybody as having TB causing agents and hence no need of worrying about it to undue fear of contracting the disease to the extent of needing to leave a TB patient alone in a separate room until the end of treatment.

#### Direct non-medical costs related to TB treatment

TB treatment is free of direct cost in Ethiopia; that is, no payment for laboratory investigations and anti TB drugs. But, there are direct non-medical costs such transportation cost and house rent costs for living in towns nearby the facilities to follow DOTs. The pastoral community lives in a scattered settlement and moves from an area to another in search of pasture for livestock. Traveling every morning for DOTs to health facilities from distant villages is not only difficult; it is also costly. Some patients prefer to rent houses near the treatment facilities; to avoid the difficulty of everyday travel to the health facilities. This is, therefore, another cost to the patients, leading them to catastrophic cost associated with TB treatment. These difficulties will negatively affect health seeking behavior of other presumptive TB patients and hence results in delayed case identification.

**Fig. 1:**
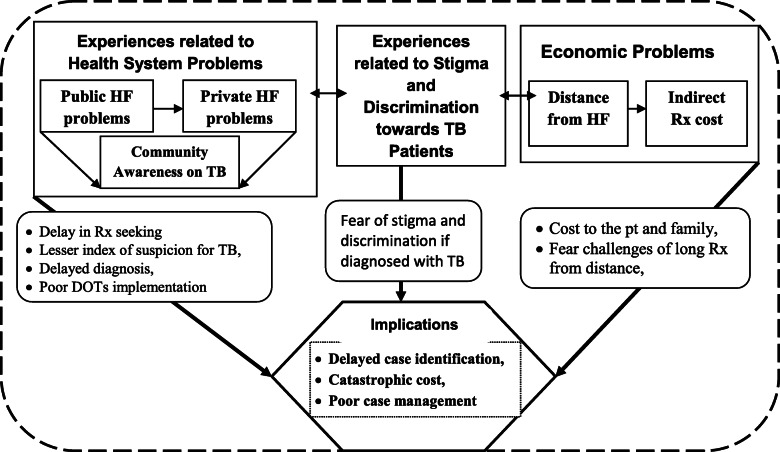
Diagrammatic depiction of the interplay among themes, sub themes and their implications

The above five categories of codes are organized and finally three themes emerged from the analysis. The three themes, interplays between sub-themes and their implications were illustrated using a diagram (Fig. 1).

## Discussion

In this study, we intended to explore lived experiences of TB patients and the implications of the experiences for timely TB case identification and case management. Accordingly, undesirable experiences related to health systems, stigma and discrimination, and socioeconomic conditions of the community in general and the patients in particular were revealed.

Low community awareness, which was apparent in this study, leads to delayed health care seeking and hence sustains transition in the community [22–24]. Private health facilities are not considered as part of the health system and not working in collaboration with public health facilities; and this lack of involvement of the private health facilities into TB prevention and control activities is entailing not only undue cost to TB patients [25]. It is contributing to delayed TB case detection [26]. Besides physical inaccessibility, some health professionals in public health facilities lack good index of suspicion for TB and some are not compliant to the national TB prevention and control guidelines. Such practices result in delayed diagnosis [24, 27] and emergence of DR TB [22, 28].

Low community awareness on TB leads to undue fear to the disease and leads to stigma and discrimination [29, 30]. Stigma and discrimination towards TB patient can both delay in treatment seeking for TB signs and symptoms and also can affect adherence to the treatment [31, 32]. In pastoralist community setting, the effect of stigma and discrimination exacerbates the problem of health care seeking for TB related signs and symptoms [24].

The other challenges, experienced by TB patients in the pastoralist community, where economic burden that may expose families of the patients to catastrophic cost [10]. Due to the inaccessibility of TB service providing health facilities, patients suffer from direct non-medical costs such as transportation cost which can lead to lost to follow up from treatment, [27, 33] poor adherence and hence development of DR TB, [23, 34] and discourages other patients from seeking care for similar signs and symptoms [25, 26]. These challenges can be minimized using different innovative and patient centered approaches such as the use of community treatment supporters [28, 35–37], modified self administered treatment methods [38]. It is good to design context appropriate and scientifically sound approaches using models such as multi-criteria decision analysis [39, 40].

### Strength and limitations of the study

Data collection by single senior data collector and reliable analysis such as checking of transcripts against audio-recordings and field notes by two independent experts to ensure rigor were among the strengths of this paper. In addition, participants from all levels of health care facilities were selected to include a wide range of patients with different experiences. Nonetheless, this study had some limitations. One of these was convenience sampling method where consecutive patients visiting health facilities were included. One of the problems with convenience sampling is that it may fail to capture important perspectives from difficult-to-reach people. But, as we included patients from all types of health care settings and the purpose of qualitative study is for in-depth understanding of the lived experiences and not for its generalization, findings of the study are important to improve TB prevention and control activities in pastoral community.

## Conclusions

In the studied pastoral community, TB patients are experiencing many challenges with the current application of ‘one size fits all’ TB prevention and control approach. These multifaceted challenges couldcause delayed case identification and sustain infection transmission. Furthermore, they lead to compromised compliance to DOTs and facilitate development of DR TB. Therefore, designing and implementing context appropriate approaches such as patient centered and modified DOTs with community treatment supporters is required to achieve the intended goals of the END TB strategy in the pastoral community.

## Data Availability

The data, both audio records and transcripts, analyzed during the current study are available from the corresponding author on reasonable request.

## Abbreviations

CEO: Chief executive officer
DOTs: Directly observed short course treatment
HF: Health facility
HIV: Human immunodeficiency virus
MDR: Multi drug resistance
Rx: Treatment
TB: Tuberculosis
